# Hemp Seed Cake as a Novel Ingredient for Dog's Diet

**DOI:** 10.3389/fvets.2021.754625

**Published:** 2021-12-14

**Authors:** Alessandro Vastolo, Sergio Iliano, Flaviana Laperuta, Saverio Pennacchio, Marina Pompameo, Monica Isabella Cutrignelli

**Affiliations:** ^1^Department of Veterinary Medicine and Animal Production, University of Naples Federico II, Naples, Italy; ^2^Municipal Kennel “La collina di Argo”, Naples, Italy; ^3^National Health Service (ASL NA1 Nord), Naples, Italy

**Keywords:** fatty acids, ALA, LA, blood parameters, nutrition, cholesterol, creatinine, hepatic biomarker

## Abstract

In the last few years, the popularity of industrial hemp and its products is increased. From a nutritional point of view, hemp and its products are rich in protein, polyunsaturated fatty acids, vitamins, and useful minerals. Nowadays, the European Commission authorizes the use of hempseed and hempseed oil co-products in animal nutrition. This study is aimed to evaluate the use of hempseed cake in dogs' nutrition, comparing the effect of the supplementation of two lipid sources: swine tallow (T-diet) and hempseed cake (H-diet). A double-blind nutritional trial was performed at a municipal kennel located in Naples. Eight crossbreed neutered dogs recognized in good health were recruited and divided into two homogeneous groups (T- vs. H-group). Both diets were analyzed for chemical composition and fatty acid profile. Blood count and biochemical profile were evaluated at recruitment (T0) and the end of the trial (T30). Oleic, palmitic, and stearic acids were the most representative fatty acids in both diets; however, the H-diet contains more than double concentration of linoleic and α-linoleic acids compared to the T-diet (*p* < 0.01). The H-diet has shown significantly (*p* < 0.01) higher peroxidation index as the only negative aspect, which could compromise its shelf-life. After 30 days of administration, the H-group has shown a significant (*p* < 0.01 and *p* < 0.05) reduction of liver and renal markers [aspartate transferase (AST), alanine transaminase (ALT), and creatinine] and cholesterol, due to the healthier fatty acid profile. Hempseed cake seems a suggestable source of polyunsaturated fatty acids for dogs considering these preliminary results.

## Introduction

In the middle of the 20th century, the *Cannabis sativa* L. hemp variety has been unfairly abandoned because of its similarity to *Cannabis indica* L., which was illegal. However, the popularity of industrial hemp and its products increased in the last few years (1). Indeed, nowadays, it can be possible to cultivate hemp varieties containing <0.2% delta-9-tetrahydrocannabinol (THC) (2). In this scenario, food production is the main purpose of cultivation (3). Hemp and its products are rich sources of protein, polyunsaturated fatty acids (PUFA), vitamins, and useful minerals as evidenced by Callaway (4). Hemp seeds contain 25–35% of oil (5), which have shown a favorable fatty acid profile. Indeed, it contains linoleic acid (LA, C18:2 n-6) and α-linolenic acid (ALA, C18:3 n-3) and most of n-6 and n-3 PUFA (6).

Regarding the use of hemp products in animal nutrition, the European Food Safety Authority (EFSA) allows the administration of hempseed and hempseed co-products as feed ingredients for all animal species with species-specific differences in diet's rate inclusion (7). Moreover, hemp oil can be used as a supplement in feed mixtures for animals as a rich source of essential fatty acids, while hempseed and hempseed cakes can be used as fat and protein sources in animals' diets (5).

The study aims to evaluate the use of hempseed cake as a source of lipids in dogs' diets. For this purpose, the effect of diets supplemented with two lipid sources (swine tallow vs. hempseed cake) was compared.

## Materials and Methods

### Animals and Diets

A double-blind nutritional trial has been performed at a municipal kennel located in Naples. The kennel was affiliated with the Department of Veterinary Medicine and Animal Production, University of Naples, Federico II, and with the National Health Service (ASL NA1 Nord). Eight crossbreed neutered dogs [age 5 ± 1.77 years, weight 16.66 ± 6.38 kg; body condition score (BCS) 4.38 ± 0.18 on a nine-point scale] were recognized in good health and recruited after clinical evaluation and hematological, biochemical, and parasitological tests. The dogs were divided into two groups [swine tallow (T-group) and hempseed cake (H-group)] homogeneous for gender, body weight, and BCS. Each dog was housed in an individual 12-m^2^ box (3 × 4) divided between a closed rest section (1 × 2) and an open area. A canned commercial diet was supplemented with the same amount (3.5% as feed) of swine tallow or hempseed cake. The latter was collected in a storage shed, which is sited in the province of Caserta (South Italy). The experimental period has lasted 40 days (10 days of adaptation and 30 days of trial) from June 8, 2021. Each diet [swine tallow (T-diet) and hempseed cake (H-diet)] was administered twice daily in a ratio of 110 kcal/kg^0.75^ of metabolizable energy (ME) (8).

### Diet Chemical Composition and Fatty Acid Profile

An aliquot of 500 g for each diet was collected twice for chemical composition (9) and fatty acid profile assessment. Total fat was previously extracted (10) from each sample and subsequently turned into methyl esters (FAMEs) by direct transesterification (11) to analyze fatty acid profiles. FAMEs were dissolved in n-hexane, filtered, and injected into a gas chromatographic system (Focus GC, Thermo Electron Corporation, Waltham, Massachusetts, USA) with a flame ionization detector (FID) for FAME assessment according to Oteri et al. (12). Fatty acid peaks were identified and quantified by comparison with an external standard composed of pure FAME mixture (Larodan Fine Chemicals, AB, Limhamnsgardens Malmo, Sweden). The peroxidation index (PI) was calculated using an equation (13), where the singular acids are reported as percentage (%): PI = (dienoic × 1) + (trienoic × 2) + (tetraenoic × 3) + (pentaenoic × 4) + (hexaenoic × 5).

### Clinical Findings

A blood sample (±10 ml) was collected to determine blood count and biochemical profile at recruitment (T0) and the end of the trial (T30), after an overnight fasting period. Blood count samples were refrigerated and quickly transported to the clinical analysis laboratory of the Department of Veterinary Medicine and Animal Production of the Federico II—University of Naples. Each blood sample was analyzed using an impedance device to perform an instrumental count (HeCo 5 Vet C, Real Time Diagnostic Systems: San Giovanni a Valdarno, Italy) after slow and constant mixing for 20 min. The following parameters were analyzed: red blood cell (RBC) count, hematocrit (HCT), hemoglobin (Hb), mean corpuscular volume (MCV), mean corpuscular hemoglobin (MHC), mean corpuscular hemoglobin concentration (MCHC), red cell distribution width (RDW), reticulocytes (Ret), reticulocyte hemoglobin content (CHr), leukocytes (Leu), lymphocytes (Lymphs), monocytes (Mono), eosinophils (Eos), basophils (Baso), and platelets (PLT).

The gel separator tubes were left at room temperature for about 15 min until a clot formed. After that, the samples were centrifuged for 10 min at a speed of 1,500 × g to obtain the serum. All procedures were performed at the kennel. The serum was stored at −80°C and sent with dry ice to a reference laboratory (Kornwestheim, Germany). There, the following parameters were determined using a Beckman biochemical analyzer (Beckman Coulter AU5400; Olympus America, Melville, NY, USA): globulin, total protein (TP), albumin (Alb), alkaline phosphatase (AP), glutamic pyruvic transaminase (GPT), alanine transaminase (ALT), gamma-glutamyl transferase (GGT), aspartate transferase (AST), glutamate dehydrogenase (GLDH), fructosamine (Fr), glucose (GLU), α-amylase, lipase (LP), sodium (Na), potassium (K), calcium (Ca), chloride (Cl), phosphorus (P), magnesium (Mg), cholesterol (CHOL), triglycerides (Tri), creatinine (CREA), urea (BUN), creatine kinase (CK), and cortisol (Cort).

### Parasitological Examination

All dogs, upon their arrival in the kennel, were screened for intestinal helminths and protozoa and, if positive, were treated with antiprotozoal and/or anthelmintic drugs, before the beginning of the study. Then, stool samples from all dogs included in the study were collected at the beginning of the study (T0), after 14 days (T14), and at the end of the trial (T30) for copromicroscopical examinations. All samples were analyzed using the FLOTAC dual technique (14) with sodium chloride (specific gravity, s.g. = 1.20) and zinc sulfate (s.g. = 1.20) as flotation solutions and an analytic sensitivity of 2 eggs/oocysts/cysts/larvae per grams of feces (EPG/OPG/CPG/LPG).

### Statistical Analysis

All statistical analyses were performed using the software JMP 14 (SAS Institute, NC, USA). *T*-test was used to analyze the differences between the fatty acid profiles of the two tested diets, while Wilcoxon non-parametric test was used to analyze the effect of the diets and time on blood count and biochemical profile due to the low number of subjects. The level of significance was α = 0.05; for all variables, mean values and standard deviation of multiple analyses are reported.

## Results

No significant differences have been observed for body weight and BCS between groups and/or periods after 1 month of diet administration. Daily feed intake for all dogs corresponded to the administered ratio (110 kcal ME/kg^0.75^). Only two dogs during the second week of the trial left about 30% of daily ratio. This variation could probably be due to the sudden change in climatic conditions (during the week: mean *T*_min_ = 22.00 ± 0.53°C; *T*_max_ = 29.57 ± 1.18°C, and humidity = 64.85% ± 3.23%) registered in the week, because, subsequently, these dogs also consumed all the administered ratio.

### Chemical Composition and Fatty Acid Profile

The H-diet has shown higher crude protein and crude fiber content and lower ether extract amount compared with the T-diet (Table 1).

**Table 1:** Chemical composition (mean and standard deviation) of administered diets.

**Nutrients (% as feed)**	Diet^*^ (*n* = 4)	**T-diet (*n* = 4)**	**H-diet (*n* = 4)**
Moisture	75.02 ± 0.25	67.35 ± 0.26	66.09 ± 0.31
CP	11.81 ± 0.18	10.54 ± 0.73	11.53 ± 0.02
CF	4.14 ± 0.20	2.99 ± 0.15	3.52 ± 0.35
EE	5.55 ± 0.56	8.91 ± 0.60	7.09 ± 0.02
Ash	3.25 ± 0.21	3.15 ± 0.07	3.70 ± 0.14
NFE	13.29 ± 0.36	6.95 ± 1.11	8.00 ± 0.22
ME (kcal/kg)^#^	1,350.3	1,286.4	1,369.4

* *Commercial diet was reported as term of comparison, and it was not statistically analyzed*.

# *Calculated according to modified Atwater's factors*.

*T-diet, tallow diet; H-diet, hemp diet; CP, crude protein; CF, crude fiber; EE, ether extract; NFE, nitrogen free extract*.

In Table 2, the fatty acid profiles of the administered diets and commercial diet are shown. In the H- and T-diets, oleic acid was the most representative fatty acid (C18:1 cis 9: 28.44% vs. 35.72% TFAs, respectively). Swine tallow shows higher (*p* < 0.01) values of saturated fatty acids (palmitic C16:0, margaric C17:0, myristic C14:0, pentadecanoic C15:0, and behenic C22:0). Moreover, γ-linolenic (GLA C18:3 n-6) and docosadienoic (C22:2 n-6) acids were higher (*p* < 0.01 and *p* < 0.05, respectively) in the diet supplemented with swine tallow than that supplemented with hempseed cake. The percentage of LA was higher in the H-diet than the T-diet (*p* < 0.01). Regarding the other fatty acids recommended for dog nutrition, α-linolenic (ALA C18:3 n-3) resulted in more than double amounts in the H-diet compared to the T-diet (*p* < 0.01). Moreover, the H-diet had higher amount of trans-vaccenic (TVA C18:1 trans 11), butyric (C4:0) (*p* < 0.01), and caproic (C6:0) (*p* < 0.05) acids than the T-diet.

**Table 2:** Fatty acid profiles (% of total fatty acids) of tested diets.

**Fatty acids**	Diet^*^	**T-diet**	**H-diet**	***P*** **-value**
C4:0	3.23 ± 0.03	2.11 ± 0.07	2.68 ± 0.02	0.0082
C6:0	0.69 ± 0.05	1.61 ± 0.03	2.37 ± 0.07	0.0246
C8:0	0.05 ± 0.007	0.04 ± 0.004	0.15 ± 0.003	0.0005
C14:0	1.97 ± 0.09	1.77 ± 0.01	0.79 ± 0.09	<0.0001
C15:0	0.17 ± 0.009	0.18 ± 0.008	0.06 ± 0.001	0.0066
C16:0	21.3 ± 0.53	23.6 ± 0.15	17.7 ± 0.04	0.0003
C17:0	3.46 ± 0.18	2.31 ± 0.08	1.71 ± 0.07	0.0069
C17:1	0.46 ± 0.02	0.46 ± 0.01	0.69 ± 0.09	0.0675
C18:1 cis6	0.22 ± 0.015	0.25 ± 0.02	0.09 ± 0.07	0.0125
C18:0	8.6 ± 0.57	15.7 ± 0.09	15.4 ± 0.10	0.0816
C18:1 trans 11 (TVA)	0.95 ± 0.06	1.23 ± 0.01	1.90 ± 0.04	0.0019
C18:1 cis 9	35.5 ± 0.033	35.7 ± 0.17	28.4 ± 0.56	0.0032
C18:1 cis 10	0.16 ± 0.03	0.29 ± 0.02	0.38 ± 0.05	0.0277
C18:1 cis 11	0.01 ± 0.007	0.02 ± 0.002	0.01 ± 0.005	0.1641
C18:2 cis n-6 (LA)	18.4 ± 0.34	9.96 ± 0.06	20.9 ± 0.09	<0.0001
C20:0	0.15 ± 0.01	0.19 ± 0.05	0.37 ± 0.03	0.0141
C18:3 n-6 (GLA)	0.04 ± 0.001	0.49 ± 0.03	0.23 ± 0.01	0.0093
C18:3 n-3 (ALA)	1.79 ± 0.01	1.30 ± 0.02	3.53 ± 0.02	<0.0001
C20:2 n-6	0.09 ± 0.07	0.32 ± 0.01	0.15 ± 0.02	0.0064
C22:0	0.27 ± 0.02	1.12 ± 0.01	0.16 ± 0.01	0.0001
C20:3 n-6	0.07 ± 0.05	0.04 ± 0.01	0.04 ± 0.02	0.9357
C20:3 n-3	0.20 ± 0.02	0.22 ± 0.01	0.61 ± 0.03	0.0026
C20:4 n-6 (AA)	0.28 ± 0.004	0.18 ± 0.02	0.12 ± 0.02	0.0691
C22:2 n-6	0.36 ± 0.01	0.29 ± 0.01	0.08 ± 0.03	0.0107
C24:0	0.06 ± 0.003	0.09 ± 0.03	0.06 ± 0.01	0.0695
C20:5 n-3 (EPA)	0.12 ± 0.002	0.15 ± 0.006	0.18 ± 0.004	0.1214
C24:1	0.02 ± 0.003	0.02 ± 0.005	0.15 ± 0.005	0.0006

* *Commercial diet was reported as term of comparison, and it was not statistically analyzed*.

*T-diet, tallow diet; H-diet, hemp diet; C4:0, butyric acid; C6:0, caproic acid; C8:0, caprylic acid; C14:0, myristic acid; C15:0, pentadecylic acid; C16:0, palmitic acid; C17:0, margaric acid; C17:1, heptadecenoic acid; C18:1 cis6, petroselinic acid; C18:0, stearic acid; C18:1 trans 11, trans vaccenic acid (TVA); C18:1 cis 9, oleic acid; C18:2 cis n-6, linoleic acid (LA); C20:0, arachidic acid; C18:3 n-6, γ-linolenic acid (GLA); C18:3 n-3, α-linoleic acid (ALA); C20:2 n-6; C22:0, behenic acid; C20:3 n-6; C20:3 n-3, dihomo γ-linolenic; C20:4 n-6, arachidonic acid(AA); C22:2 n-6, docosadienoic acid; C24:0, lignoceric acid; C20:5 n-3, eicosapentenoic (EPA)*.

The differences between fatty acid profiles of the diets have been shown in Table 3. As observed previously, the T-diet had higher levels of saturated (SFA) and monounsaturated (MUFA) fatty acids (*p* < 0.01), while the H-diet had higher (*p* < 0.01) levels of PUFA of both categories (n-6 and n-3). Moreover, the H-diet resulted in significantly lower n-6/n-3, LA/ALA, and AA/eicosapentenoic acid (EPA) ratios (*p* < 0.01 and *p* < 0.05, respectively). Otherwise, the T-diet had a lower (*p* < 0.01) PUFA/SFA ratio. The PI was significantly (*p* < 0.01) higher in the H-diet compared to the T-diet.

**Table 3:** Categories of fatty acids (% of total fatty acids) of the administered diets.

**Categories**	Diet^*^	**T-diet**	**H-diet**	***P*** **-value**
SFA	40.5 ± 0.53	48.9 ± 0.19	41.5 ± 0.10	0.0004
MUFA	37.8 ± 0.42	38.3 ± 0.18	31.9 ± 0.60	0.0048
PUFA	21.2 ± 0.38	12.8 ± 0.09	25.9 ± 0.01	<0.0001
n-6	19.2 ± 0.36	11.3 ± 0.09	21.6 ± 0.002	<0.0001
n-3	2.01 ± 0.01	1.57 ± 0.001	4.32 ± 0.01	<0.0001
PUFA/SFA	0.52 ± 0.002	0.26 ± 0.0008	0.62 ± 0.002	<0.0001
n-6/n-3	9.57 ± 0.11	7.18 ± 0.05	4.99 ± 0.01	0.0003
LA/ALA	10.3 ± 0.10	7.18 ± 0.04	5.93 ± 0.03	0.0004
AA/EPA	13.3 ± 1.35	3.47 ± 0.10	0.69 ± 0.03	0.0418
PI	82.5 ± 0.35	66.1 ± 0.02	88.2 ± 0.58	0.0004

* *Commercial diet was reported only as data, and it was not statically analyzed*.

*T-diet, tallow diet; H-diet, hemp diet; SFA, saturated fatty acids; MUFA, monounsaturated fatty acids; PUFA, polyunsaturated fatty acids; LA/ALA, linoleic acid/α-linolenic acid; AA/EPA, arachidonic acid/eicosapentaenoic acid; PI, peroxidation index*.

### Clinical Findings

All values of blood count (data not shown) fall in the physiological range for the species. No significant differences were observed between groups and sampling periods in blood count parameters. The number of reticulocytes was the only one that was significantly lower in the animals fed with the T-diet than in those fed with the H-diet (38.8 ± 1.15 vs. 70.1 ± 1.30 K/μl; *p* < 0.05).

All biochemical parameters were within the normal reference ranges for dogs. For brevity, only statically significant results were reported (Table 4). Comparing the groups, animals fed with the T-diet showed higher levels of AST and CHOL (*p* < 0.01) and CREA, ALT, and CK (*p* < 0.05). Considering the time effect, AP significantly decreased during the trial (*p* < 0.01). Otherwise, after 30 days, bilirubin (BIL), glucose, and Cl were higher (*p* < 0.05) than at the beginning of the trial.

**Table 4:** Main biochemical parameters.

**Parameters**	**Units**	**T-group**	**H-group**	**T0**	**T30**	***P*** **-value**	
						**Diet effect**	**Time effect**
AP	U/l	35.3 ± 4.0	25.6 ± 2.0	51.0 ± 9.2	19.4 ± 3.55	0.7144	0.0080
CREA	μmol/l	103 ± 8.55	87.0 ± 11.0	96.8 ± 12.7	95.2 ± 13.6	0.0270	0.8538
AST	U/l	49.6 ± 6.66	30.7 ± 3.01	40.6 ± 11.0	38.2 ± 11.8	0.0058	0.5200
ALT	U/l	43.3 ± 4.50	29.0 ± 3.56	38.2 ± 6.98	37.0 ± 10.4	0.0101	0.9163
BIL	μmol/l	3.12 ± 0.28	2.19 ± 0.83	1.70 ± 0.92	3.19 ± 1.09	0.1483	0.0136
CHOL	mmol/l	5.59 ± 0.45	4.41 ± 0.63	4.82 ± 0.77	5.14 ± 0.85	0.0057	0.4747
GLU	mmol/l	5.04 ± 0.48	5.13 ± 0.39	4.77 ± 0.36	5.33 ± 0.30	0.9489	0.0197
CK	mmol/l	143 ± 10.4	82.0 ± 5.9	116 ± 11.8	121 ± 12.7	0.0105	0.9168
Cl	mmol/l	113 ± 3.25	112 ± 3.27	110 ± 2.41	114 ± 2.54	0.6976	0.0177

*AP, alkaline phosphatase; CREA, creatinine; AST, aspartate transaminase; ALT, alanine aminotransferase; BIL, bilirubin; CHOL, cholesterol; GLU, glucose; CK, creatine kinase; CL, chloride*.

### Parasitological Examination

All dogs tested negative for all endoparasites throughout the trial.

## Discussion

### Chemical Composition and Fatty Acid Profile

Both diets guarantee minimal requirement of macronutrients suggested for adult dogs located in a kennel (8). The registered value of feed intake seems to indicate that the diets were palatable. Indeed, the temporary reduction of feed intake shown by two dogs, one per group, during the first week of July seems related to the climatic condition variation.

Regarding the fatty acid profile of the two tested diets, hempseed cake supplementation seems to increase the percentage of PUFA into the commercial diet, especially linoleic (LA C18:2 n-6) and α-linoleic (ALA C18:3 n-3) acids. The increase could be due to the high percentage of polyunsaturated fatty acids in hempseed cake (15, 16). The swine tallow diet appears to be influenced by the chemical composition of the supplementation like the H-diet. The T-diet has shown a high percentage of SFA and MUFA. The saturated fat present in tallow could be useful for the companion animals as source of energy to work, regulate body temperature, growth and reproduction. Moreover, these fats could be stored in adipose tissues for future mobilization and used for energy when needed (17). Otherwise, hempseed cake is rich in LA and ALA (18, 19), which are essential for dogs considering that this species cannot synthesize LA and ALA *ex novo*. Linoleic acid (LA 18:2 n-6) was the only essential fatty acid listed for dogs by the National Research Council until recently (20). Both types of omega fatty acids can be converted to longer chain polyunsaturated fatty acids that have additional essential functions (17). Particularly, ALA could be converted to EPA and docosahexaenoic (DHA) acids, which are necessary for dog. Indeed, n-6 and n-3 fatty acids operate as precursors of the eicosanoids, which are important to the cell functions (17). Consequently, it is necessary to include LA and ALA in dogs' diets (21). The differences between fatty acid categories affected the ratio between categories, and these changes could influence human (13) and animal (20) health. The hempseed cake diet has shown an n-6/n-3 ratio lower than 5:1, which has been claimed as ideal for humans and dogs (22). Polyunsaturated fatty acids are very important for the development of the nervous system, and an optimal dietary n-6/n-3 fatty acid ratio reduces the incidence of some diseases, such as cancer and sudden cardiac death (23). Moreover, it is important to have adequate amounts of DHA and EPA, which are useful for neurologic development, particularly during the early stage of growth. In our case, the level of ALA in the hempseed cake diet is sufficient to allow the conversion to EPA and DHA. In this regard, Bauer (24) observed that puppies that suckle ALA-rich milk appear to accumulate DHA in their plasma for a short time prior to weaning. Furthermore, the presence of PUFA in the diet influences growth and health status of dogs, helps to increase general metabolic rates, and may promote the burning of fat (25).

The presence of n-3 PUFA in the diet allows obtaining other benefits such as anti-inflammatory and potentially anti-thrombotic properties (26). In particular, a higher amount of n-3 fatty acids could have a positive effect, especially in dogs with pruritus and other inflammatory diseases (27). Indeed, essential fatty acids from n-6 and n-3 families are proven to have anti-inflammatory effects and immunomodulating properties on skin diseases (28). Furthermore, dietary PUFA seem to affect animal behavioral changes. Indeed, the dopaminergic and serotonergic systems in the brain are known to play important roles in learning, emotions, and impulse control. Both these systems are known to be influenced by PUFA; thus, it is necessary to feed animals with the right amount of polyunsaturated fatty acids in the diet (29).

However, the higher percentage of PUFA could limit diet shelf-life as demonstrated by the increased peroxidation index of the H-diet with respect to the T-diet. Diets that contain a high amount of PUFA must be protected from light and high temperatures that could cause their oxidation during the production process and storage (23).

### Clinical Findings

All dogs were in good health at the end of the trial as evidenced by clinical evaluation (body weight and BCS) and blood parameters. The increase of chloride and AP could be difficult to explain as well as the rise in reticulocytes in the dogs fed with the H-diet.

The animals in the H-group have shown lower levels of cholesterol, AST, ALT, creatinine, and CK compared to those fed with the T-diet. These results could suggest a beneficial effect of diet supplemented with hempseed cake due to the high amount of PUFA, particularly the linoleic and α-linolenic acids. In this regard, dietary supplementation with PUFA, in particular ALA, may be a useful addition to treatment in renal disease, rheumatoid arthritis, cutaneous inflammatory disorders, thromboembolic disease, and autoimmune diseases (29).

All liver markers (AST and ALT) and cholesterol decreased by the presence of both n-6 and n-3 PUFA. Furthermore, these results partially agreed with the one's obtained by Welch-White et al. (30), who observed the reduction of several serum parameters, such as AST, ALT, GGT, cholesterol, and triglycerides, with the increasing level of PUFAs administered with the diets in rats. Moreover, Kaushal et al. (31) evidenced the specific anti-hypercholesterolemic effects of hemp seed related to redox-sensitive modulation of inflammatory pathways in rats, which prevent fat deposition into the liver and arterial lumen.

The higher PUFA concentration of the H-diet could have also caused the significant reduction of creatinine, considered a biomarker of renal function.

Brown et al. (32) observed in short-term studies in dogs with naturally occurring renal disease that supplementation with n-6 PUFA led to an increased glomerular filtration rate (GFR). The same authors detected that dietary supplementation with n-3 PUFA decreased glomerular capillary pressure and seems to be renoprotective. On that basis, n-3 PUFA, particularly EPA and DHA, and their precursor (ALA) could be useful in the management of dogs with naturally occurring renal diseases.

It is possible to hypothesize that both lipidic supplementations of the diet had modified the dogs' serum lipidomic profile as suggested by Boretti et al. (33), even if in this study the lipidomic serum was not analyzed.

### Parasitological Examination

All dogs screened tested negative for intestinal helminths and protozoa during the entire trial. That may be due to a regular monitoring and subsequent treatment of dogs as suggested by the guidelines of the European Scientific Counsel Companion Animal Parasites (34, 35). Moreover, good management and hygiene practices (36, 37) were used as a complementary program to achieve effective control of endoparasitosis in dogs.

## Conclusion

It seems possible to suggest the use of this co-product as a polyunsaturated fatty acid source for dog nutrition from these preliminary results, despite the lack of proximate analysis data of hempseed cake. Even if the increased peroxidation index of the diet supplemented with hemp co-product seems to indicate that, further studies are necessary to better identify the strategies to prevent the oxidation of hempseed cake during pet-food production and storage.

Despite the low number of recruited subjects and the lack of lipidomic serum analysis, the improvement of several hematological parameters, such as cholesterol, renal, and hepatic biomarkers, confirmed that the addition of hempseed cake and its enriched PUFA profile are safe for dogs to consume.

## Data Availability Statement

The raw data supporting the conclusions of this article will be made available by the authors, without undue reservation.

## Ethics Statement

All the procedures used in the study have been approved by the Ethics Committee for the care and use of animals of the University of Naples Federico II in accordance with local and national regulations and guidelines (Legislative Decree 26 of 04/03/2014).

## Author Contributions

MC conceptualized and supervised the study. AV, FL, SP, and SI conducted the formal analysis. SI and AV contributed in the methodology and data curation and wrote the original draft. AV conducted the statistical analysis. MP and FL conducted clinical visitation. MC and MP reviewed and edited the manuscript. All authors contributed to the article and approved the submitted version.

## Funding

This trial was partially supported by Farmina Pet Food (Nola, Italia) and by DMVPA funding.

## Conflict of Interest

The authors declare that the research was conducted in the absence of any commercial or financial relationships that could be construed as a potential conflict of interest.

## Publisher's Note

All claims expressed in this article are solely those of the authors and do not necessarily represent those of their affiliated organizations, or those of the publisher, the editors and the reviewers. Any product that may be evaluated in this article, or claim that may be made by its manufacturer, is not guaranteed or endorsed by the publisher.
